# RNA Profiling Analysis of the Serum Exosomes Derived from Patients with Active and Latent *Mycobacterium tuberculosis* Infection

**DOI:** 10.3389/fmicb.2017.01051

**Published:** 2017-06-12

**Authors:** Lingna Lv, Cuidan Li, Xiuli Zhang, Nan Ding, Tianshu Cao, Xinmiao Jia, Jinghui Wang, Liping Pan, Hongyan Jia, Zihui Li, Ju Zhang, Fei Chen, Zongde Zhang

**Affiliations:** ^1^Beijing Key Laboratory for Drug Resistant Tuberculosis Research, Beijing Tuberculosis and Thoracic Tumor Research Institute, Beijing Chest Hospital, Capital Medical UniversityBeijing, China; ^2^CAS Key Laboratory of Genome Science and Information, Beijing Institute of Genomics, Chinese Academy of SciencesBeijing, China; ^3^University of Chinese Academy of SciencesBeijing, China; ^4^Collaborative Innovation Center for Genetics and DevelopmentShanghai, China; ^5^Sino-Danish College, University of Chinese Academy of SciencesBeijing, China

**Keywords:** tuberculosis (TB), *Mycobacterium tuberculosis* (*Mtb*), exosome, RNA sequencing, latent tuberculosis infection (LTBI), transcriptome, biomarker, IPA

## Abstract

Tuberculosis (TB) has exceeded HIV as the most lethal infectious disease globally for two consecutive years. Moreover, one third of the world’s population is estimated to have latent tuberculosis infection (LTBI). This is mainly because of difficulties associated with diagnosis and treatment for both TB and LTBI patients. Exosomes provide a promising research tool for TB diagnosis and treatment because they are released from various cells containing valuable biochemical information related to disease. In this study, we performed RNA-sequencing analysis on exosomes derived from clinical specimens of healthy controls (HC), active tuberculosis (ATB), and LTBI patients. Our results revealed the distinct gene expression profiles of the exosomes from LTBI and ATB patients. (1) We identified many distinct up-regulated and down-regulated differentially expressed genes (DEGs) in LTBI and ATB samples, and further screened the top-20 DEGs which might provide a potential panel for differentiation of HC, LTBI, and ATB. (2) We classified all the DEGs into six expression patterns, screened the top-20 genes in each pattern, and mainly focused on those highly expressed in LTBI and ATB. (3) Some *Mycobacterium tuberculosis* (*Mtb*) RNAs were only enriched in the exosomes of LTBI samples. (4) Pathway and function analysis further indicated down-regulated signaling pathways/immune response and up-regulated apoptosis/necrosis. Our findings indicate the selective packaging of RNA cargoes into exosomes under different stages of *Mtb* infection, while facilitating the development of potential targets for the diagnosis, prevention and treatment of tuberculosis.

## Introduction

According to the 2015 and 2016 WHO reports, tuberculosis has exceeded HIV as the most lethal infectious disease globally for two consecutive years (2014 and 2015). This is mainly because of difficulties associated with diagnosis and treatment of TB patients. In addition, elimination of tuberculosis has been partly prevented by the ability of *Mtb* to remain dormant in the human body for years without causing disease, a state referred to as LTBI (Velayati et al., 2016). An estimated 2 billion people are latently infected with *Mtb*. Of these, 10% of infected individuals will develop active TB during their lifetime (Tufarielloa et al., 2003). Although the majority of infected individuals display no symptoms of disease, they develop a strong acquired immune response to the pathogen (Gideon and Flynn, 2011). Thus, early diagnosis of LTBI patients plays a crucial and important role in reducing morbidity, while also preventing onward transmission to susceptible individuals.

Exosomes provide a promising research tool for TB diagnosis and treatment because they are released from various cells containing valuable biochemical information (proteins, lipids, and nucleic acids) relating to disease, while facilitating cell-cell communication by shuttling various molecules from donor to recipient cells. These membrane-enclosed vesicles (range in size from 30 to 150 nm) play important roles in signal transduction (e.g., immune regulation) (Alipoor et al., 2016), material transportation (nucleic acids, proteins, lipids, and other biochemicals) (Théry, 2011), and cellular “trash bags” for elimination of excess intracellular substances (Subramanian et al., 2016).

Proteomic analysis has revealed many host proteins in addition to 41 *Mtb* proteins within exosomes secreted by macrophages infected with either live *Mtb* or *Mtb* culture filtrate (Giri et al., 2010). Subsequent studies identified 20 *Mtb* proteins in exosomes isolated from the serum of TB patients, including the antigens 85b, BfrB, GlcB, and Mpt64 (Kruh-Garcia et al., 2014). In addition, exosomes derived from the macrophages infected with *Mycobacterium avium* contained bacterial pathogenic glycopeptidolipids (Bhatnagar and Schorey, 2007). The extracellular exosomes carrying mycobacterial constituents have been reported to affect recipient cells by either silencing or promoting the immune responses. For example, exosomes released from infected macrophages stimulated naïve macrophages to produce proinflammatory TNF-α, RANTES, and inducible nitric oxide synthase (iNOS) (Bhatnagar and Schorey, 2007; Bhatnagar et al., 2007). Conversely, in some cases, exosomes released from *Mtb*-infected cells exhibited an inhibitory effect on IFN-γ-mediated activation of naïve macrophages (Singh et al., 2011).

Exosomes have also been found to contain various RNAs, including mRNA, rRNA, microRNA, and long non-coding RNA (lncRNA) (Gusachenko et al., 2013). Previous studies have indicated that the exosomes could transfer mRNAs to exchange phenotypic features between cells (Valadi et al., 2007). Singh et al. (2015) discovered some potential biomarkers (human miRNAs and mRNAs as well as mycobacterial RNAs in exosomes) that could be used to detect TB in patients. However, systematic exosomal RNA sequencing analysis in human clinical specimens of ATB and LTBI patients is still lacking.

In this research, we performed systematic RNA profiling analysis of the exosomal RNAs derived from the serum samples of HC, LTBI, and ATB individuals. Our results revealed distinct gene expression panels and patterns of the exosomes, indicating the selective packaging of RNA cargoes into exosomes under different physiological status. Pathway and functional analysis further indicated a gradual increase in deteriorated healthy signals in LTBI and ATB samples, including down-regulated signaling pathways/immune response, and up-regulated apoptosis/necrosis. These results provide key information on exosomes during the *Mtb* infectious process, and provide insight into the development of potential biomarkers using exosomal RNAs in TB diagnosis.

## Materials and Methods

### Study Participants

This study was carried out in accordance with the recommendations of the Helsinki Declaration and its later amendments or comparable ethical standards, the Ethics Committee of the Beijing Chest Hospital, Capital Medical University with written informed consent from all subjects. All subjects gave written informed consent in accordance with the Declaration of Helsinki. The protocol was approved by the Ethics Committee of the Beijing Chest Hospital, Capital Medical University. All participants were at least 18 years old, HIV-negative and written, informed consent was obtained from these patients. TB patients were classified by their clinical presentation being consistent with TB infection, a positive *Mtb* culture, and a positive smear. Patients were excluded if they had a previous TB history or had received anti-TB therapy before enrollment. Latently infected subjects were defined as having a positive TST and IGRA using T-SPOT.TB (Oxford Immunotec, Abingdon, United Kingdom), normal chest computed tomography (CT), absence of clinical symptoms or evidence of active TB and other non-tuberculosis respiratory infections. The TST/IGRA two-step strategy is used because confirmatory IGRA is able to highly reduce the false positivity rate due to BCG vaccination or NTM infection in the initial TST. Healthy uninfected controls were enrolled with negative TST and T-SPOT.TB tests, normal chest CT and no clinical evidence of any diseases. The participant demographic information is shown in **Table 1**.

**Table 1:** Clinical data of the participants.

	HC 1	HC 2	LTBI 1	LTBI 2	ATB 1	ATB 2
*N*	15	15	15	15	15	15
Male/Female	9/6	7/8	6/9	7/8	11/4	12/3
Mean age ±*SD* (y)	41.1 ± 8.2	43.4 ± 8.6	42.2 ± 8.2	40.0 ± 7.9	41.5 ± 8.9	42.6 ± 9.6
Age range (y)	22–56	24–59	25–55	25–58	20–65	19–65
Smokers/Non-smokers	1/14	5/10	3/12	5/10	5/10	6/9

*HC, healthy control; LTBI, latent tuberculosis infection; ATB, active tuberculosis. Serum exosomes were obtained from the two biological replicate pools (*n* = 15) for the healthy control (HC), LTBI, and ATB samples.*

### Sample Preparation

A total of 90 serum samples were collected. The serum samples were grouped according to the clinical cohort as ATB, LTBI, and healthy control (HC). Serum was obtained from each participant and then pooled based on the group (pooled *n* = 15 for HC, LTBI, and ATB, respectively; 1 mL of serum in each pooled sample). For each group, two biological replicate pools were prepared (i.e., *n* = 30 patients in total for HC, LTBI, and ATB, respectively).

### Exosome Isolation and RNA Extraction

Exosome isolation from pooled serum samples was conducted as previously described. Briefly, the cell debris were removed by differential centrifugation at 1000 × *g* for 10 min at 4°C, and 16,500 × *g* for 30 min at 4°C, followed by ultrafiltration (through a 0.22 μm filter; Millipore, Billerica, MA, United States). Then, the exosome pellet was obtained by ultracentrifugation at 120,000 × *g* for 2 h and washed with PBS (Supplementary Figure 1). Isolated exosomes were immediately used for total RNA extraction using RNAiso-Plus (TaKaRa, Dalian, China) according to the manufacturer’s instructions. RNA concentration was measured using Qubit^®^ RNA Assay Kit in Qubit^®^ 2.0 Fluorometer (Life Technologies, Camarillo, CA, United States). RNA integrity was assessed using the RNA Nano 6000 Assay Kit of the Agilent Bioanalyzer 2100 system (Agilent Technologies, Santa Clara, CA, United States).

### Sequencing and Data Processing

Sequencing libraries were generated using NEBNext^®^ Ultra^TM^ Directional RNA Library Prep Kit for Illumina^®^ (NEB, Ipswich, MA, United States) following manufacturer’s recommendations and index codes were added to attribute sequences to each sample. The library quality was assessed on the Agilent Bioanalyzer 2100 system (Agilent Technologies, Santa Clara, CA, United States). The RNA libraries were sequenced on the Illumina Hiseq 2500 Genome Analyzer platform in pair-end mode.

For each sample, the mRNA sequencing reads were aligned to the UCSC human reference genome (hg38) using Tophat (version 2.0.9) (Langmead et al., 2009; Trapnell et al., 2009) with default parameters. The aligned reads were further subjected to Cufflinks (version 2.2.1) (Trapnell et al., 2010) software for assembling transcripts with the parameter “-M” for filtering reads mapped on ribosomal RNAs. DEGs were generated using Cuffdiff (version 2.2.1) (Trapnell et al., 2010). The GTF annotation file was downloaded from the GENCODE (version 25) website for transcript assembling and gene annotation. Unsupervised hierarchical clustering of genome-wide expression profiles (read count > 1) was performed using the pheatmap (R software). Gene Ontology (GO) function enrichment was generated by both DAVID (version 6.8) (Huang et al., 2009a,b). We also performed core analysis and comparison analysis using the Ingenuity Pathway Analysis (IPA) software^1^ to obtain canonical pathway and disease and bio functions, which were evaluated by *p*-value (*p*-value < 0.05) and *Z*-score (*Z*-score ≠ 0). Sequence data is now available on the Gene Expression Omnibus (GEO; accession number: GSE94907).

## Results

### Expression Profiles of Serum Exosomes in HC, LTBI, and ATB Individuals

**FIGURE 1:**
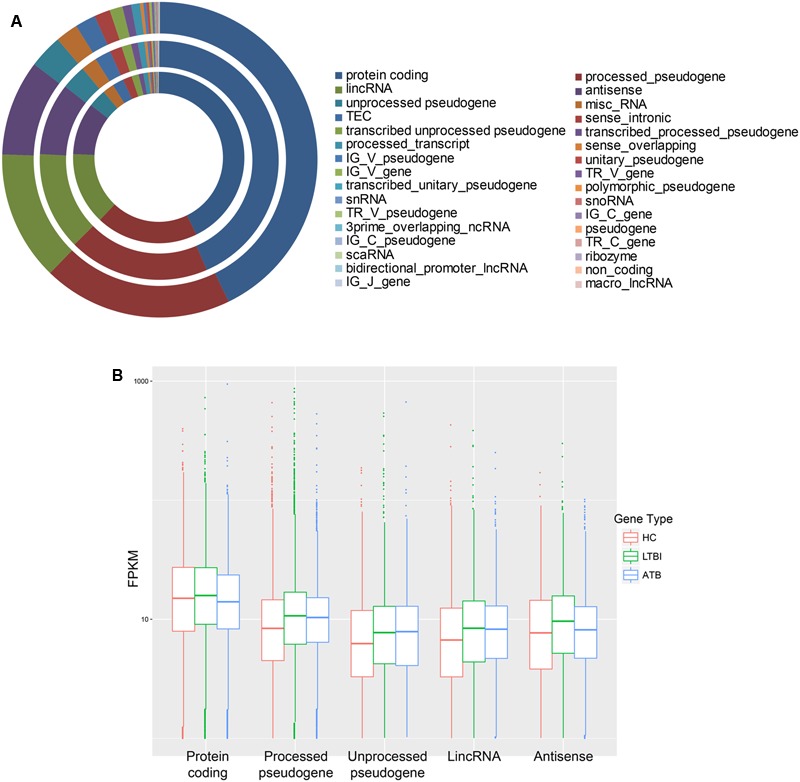
General gene expression profiles among healthy control (HC), LTBI, and ATB samples. **(A)** Many types of RNAs were secreted into the exosomes (shown in different colors). They also shared a similar proportion of gene number in each sample (circles from outer to inner represent HC, LTBI, and ATB). **(B)** The FPKM values of top-five types of RNAs in the three samples.

By mapping to the human reference genome (hg38), we obtained the gene expression profiles of serum exosomes. A total of 44187 (HC), 43428 (LTBI), and 44261 (ATB) expressed genes were identified, of which 18913, 18882, and 18926 coding genes were obtained from HC, LTBI, and ATB patients, respectively (Supplementary Table 1). As shown in **Figure 1A**, many types of RNAs, including mRNAs, pseudogenes, and lncRNAs, were secreted into the exosomes, which shared a similar proportion of genes in each sample (HC, LTBI, and ATB).

As for the expression level, the coding genes of LTBI had higher expression levels than those of HC and ATB (**Figure 1B**). On the contrary, pseudogenes had lower expression levels in serum exosomes of HC than in LTBI and ATB samples. Specifically, unprocessed pseudogenes had slightly higher expression levels in ATB patients than in LTBI patients. In addition, lncRNAs in serum exosomes of LTBI exhibited higher expression levels compared with HC and ATB samples. Although little is known of how lncRNAs are packaged into exosomes, previous studies have suggested that exosomal lncRNAs had phenotypic effects within the recipient cells (Kogure et al., 2013).

**FIGURE 2:**
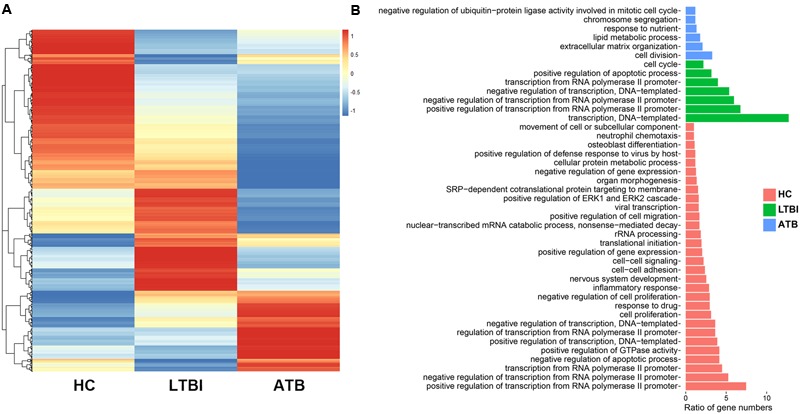
Profiles of DEGs among HC, LTBI, and ATB samples. **(A)** Heatmap of DEGs among the three samples. **(B)** The Gene Ontology (GO) biological process categories of the top 30 percentage of the DEGs in each samples.

To obtain the differential expression profiles among the serum exosomes in the HC, LTBI, and ATB individuals, we selected the DEGs by pairwise comparison (fold change ≥ 2 and *p*-value < 0.05), and plotted a heatmap (**Figure 2A**). The results revealed the distinct gene expression profiles of the exosomes for the HC, LTBI, and ATB patients: 1188 genes were highly expressed in HC samples compared with LTBI and ATB samples (the left column of **Figure 2A**). In contrast to the HC and ATB samples, 1020 genes (the middle column) were highly expressed in LTBI samples, while 681 genes (the right column) were highly expressed in ATB samples compared with the other two samples.

Further GO functional analysis indicated different enrichments of GO categories in the three samples (**Figure 2B**). Among them, 12 and 14 genes in ATB samples displayed enrichments in “lipid metabolism” and “extracellular matrix organization” GO categories. Previous studies have indicated that *Mtb* can survive in macrophages using lipid from host cells (Ouellet et al., 2011). Therefore, the up-regulation of lipid metabolic genes in ATB exosomes might accelerate the transfer of signaling substances to facilitate the survival of *Mtb* in ATB patients. On the other hand, three of the 14 up-regulated extracellular matrix genes coded for three collagens (19α1, 1α2, and 11α1), which have been reported to help form granuloma (Kaarteenaho-Wiik et al., 2007; Shammari et al., 2015).

### Expression Panels of Serum Exosomes in HC, LTBI, and ATB Individuals

**FIGURE 3:**
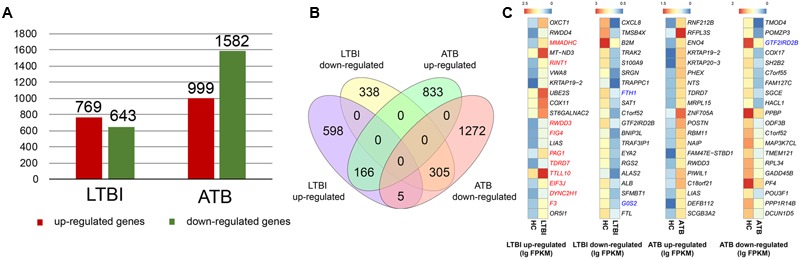
Expression panels of serum exosomes for the LTBI and ATB samples. **(A)** The bar plot showed the numbers of DEGs in the LTBI and ATB compared with HC. Red bars indicated the number of up-regulated genes in each sample, while the green bars indicated the number of down-regulated genes in each sample. **(B)** Venn diagram of DEGs showing the relatively distinct expression panels between the ATB and LTBI samples. **(C)** Heatmaps of top-20 DEGs in LTBI and ATB: colors indicate the estimate of base 10 log ratio of expression levels that ranges from blue (low expression) to red (high expression). The three genes (labeled with blue color) showed the same down-regulated expression trends in both peripheral blood mononuclear cell (PBMC) and serum exosomes. The 10 up-regulated genes in LTBI (labeled with red color) were enriched in GO functions of protein binding or protein processing.

In comparison with the HC samples, we identified 769 up-regulated genes and 643 down-regulated genes in the LTBI individuals, while these numbers increased to 999 (up-regulated) and 1582 (down-regulated) in ATB individuals (**Figure 3A** and Supplementary Table 2). To further investigate the detailed differences in DEGs between LTBI and ATB samples, we overlapped these using a Venn diagram (**Figure 3B**). They exhibited relatively distinct expression panels: 598 and 833 genes were only up-regulated and expressed in LTBI and ATB, respectively; 338 and 1272 genes were uniquely down-regulated and expressed in LTBI and ATB, respectively; while 5 genes were up-regulated and expressed in LTBI but down-regulated and expressed in ATB. In addition, there was a small portion of DEGs (fold change > 2 and *p*-value < 0.05) in ATB and LTBI that shared similar expression panels (166: up-regulation; 305: down-regulation).

To illustrate the expression panels, the top-20 DEGs (*p* < 0.05) were selected (**Figure 3C**), which might provide a potential panel for the differentiation of HC, LTBI, and ATB samples. Due to inadequate studies on serum exosomes of LTBI and ATB individuals, we compared our results with other studies performed in peripheral blood mononuclear cells (PBMCs) (Lee et al., 2016). Three genes (*FTH1* and *GOS2* in LTBI; *GTF2IRD2B* in ATB) shared the same down-regulated expression trends in PBMCs (Lee et al., 2016). However, there were some unmatched results, which increased in serum exosomes of LTBI but decreased in the serum cells of LTBI, e.g., *RINT1* (Lee et al., 2016). This suggested that the contents of exosomes showed selective enrichment and secretion (Hannafon and Ding, 2013). Incidentally, 10 of the 20 up-regulated genes in LTBI were enriched in GO functions of protein binding or protein processing.

### Expression Patterns of Serum Exosomes in HC, LTBI, and ATB Individuals

**FIGURE 4:**
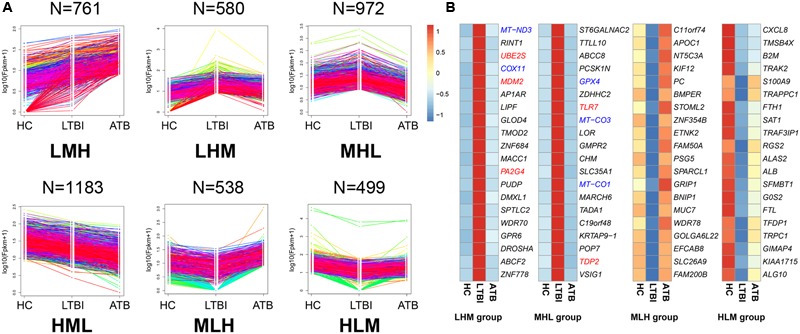
The expression patterns of DEGs among the HC, LTBI, and ATB samples. **(A)** Six patterns were determined based on FPKM values, including LMH, LHM, MHL, HML, MLH, and HLM. (L, low expression level; M, medium expression level; H, high expression level; N, the gene numbers of each pattern). **(B)** Heatmaps of top-20 high expressed and low expressed DEGs (LHM, MHL, MLH, and HLM patterns in the LTBI samples): colors indicate the estimate of base 10 log ratio of expression levels that ranges from blue (low expression) to red (high expression). The five genes (PA2G4, MDM2, UBE2S, TLR7, and TDP2, labeled with red color) in LHM and MHL groups were enriched in “cell cycle” functional category. The five genes (COX11, MT-ND3, MT-CO1, MT-CO3, and GPX4, labeled with blue color) were enriched in “mitochondria disorder” functional category.

We classified all DEGs into six expression patterns according to the gene expression trends among the three samples (**Figure 4A**, **Table 2**, and Supplementary Table 3).

**Table 2:** The number of genes for six expression patterns.

Group	Number of genes
LMH-group	761
HML-group	1,183
LHM-group	580
MHL-group	972
MLH-group	538
HLM-group	499

*^∗^ L, low expression level; M, medium expression level; H, high expression level.*

We further screened the top-20 genes in each pattern (**Figure 4B** and Supplementary Figure 2), especially those that were highly expressed in LTBI patients, which may provide potential targets of clinical diagnosis for these individuals. Five genes (*COX11, MT-ND3, MT-CO1, MT-CO3*, and *GPX4*) (Lee et al., 2009; Behar et al., 2011) with roles in mitochondrial disorder were highly expressed in LTBI patients. Previous studies have demonstrated that the integrity of the mitochondrial membrane is closely related to apoptosis, and is an effective anti-mycobacterial host defense mechanism (Ma et al., 2016). Interestingly, five genes (*PA2G4, MDM2, UBE2S, TLR7*, and *TDP2*) in both the LHM and MHL groups were identified as being enriched in the “cell cycle” functional category. As for the ATB highly expressed groups (Supplementary Figure 2), 12 genes displayed an increased trend and were in agreement with the previous studies (Pacis et al., 2015) (Supplementary Table 4). Among which, *ACSL4* and *CLU* were related with lipid metabolic process (Kuch et al., 2014; Park et al., 2014), and *MMRN1* and *POSTN* were relevant to cell adhesion (Adam et al., 2005; Michaylira et al., 2010). These results suggested that the top-20 genes for each pattern might provide potential molecular targets for the prevention, diagnosis, and treatment of LTBI and ATB individuals.

### *Mtb* RNAs Were Detected in LTBI Patients

To investigate whether *Mtb* genes were secreted in serum exosomes, the sequencing data was mapped to the *Mtb* reference genome (NC00962). We identified 2, 1101, and 3 *Mtb* genes in HC, LTBI, and ATB samples, respectively. The results showed 2 *Mtb* rRNA genes (*rrs* and *rrl*) and 3 *Mtb* genes (*rrs, rrl*, and *Rv2917*) in HC and ATB samples, which were consistent with other studies where a few *Mtb* peptides were observed in ATB and HC individuals (Gutkin et al., 2016).

**FIGURE 5:**
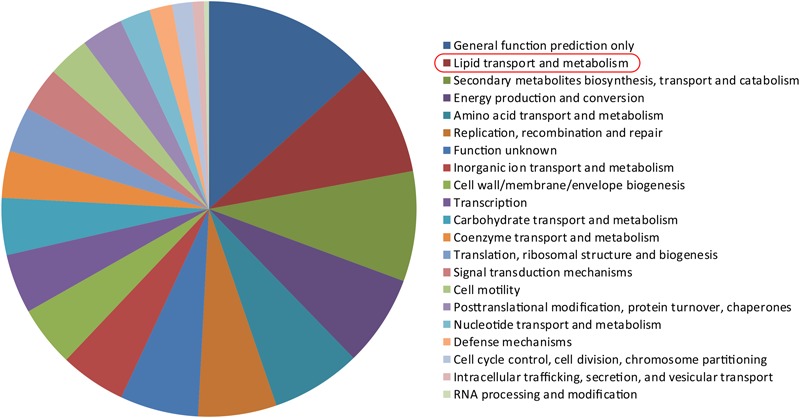
*Mtb* RNAs detected in HC, LTBI, and ATB. We identified 2, 1 101, and 3 *Mtb* genes in HC, LTBI, and ATB samples, respectively. The pie plot on the right panel indicated Cluster of Orthologous Group (COG) analysis on the 1 101 *Mtb* genes in LTBI. A enrichment on COG category I “lipid transport and metabolism” was observed.

Importantly, many *Mtb* genes (1101) were discovered in LTBI exosomes. We further performed Cluster of Orthologous Group (COG) analysis for these and found a significant enrichment in COG category I “lipid transport and metabolism” (**Figure 5**). As for the expression of *Mtb* genes, we listed the top-20 expressed genes (evaluated by reads number, Supplementary Table 5), most of which were crucial genes for immunogenicity and virulence of *Mtb* (these included pks family, PPE family, and transpoases). Previous studies also indicated numerous *Mtb* proteins in exosomes from serum samples of LTBI individuals using Multiple Reaction Monitoring-Mass Spectrometry (MRM-MS) (Sinsimer et al., 2008). Comparing the results of the aforementioned study with ours, we found that eight *Mtb* genes were detected both in forms of protein and RNA (Supplementary Table 6). These results suggested that some *Mtb* cells in LTBI patients were disrupted and that many *Mtb* RNAs and proteins were exocytosed into the serum exosomes, although it was known that the *Mtb* cells in LTBI individuals were in a dormant state (Gengenbacher and Kaufmann, 2012).

## Discussion

In this research, we revealed distinct gene expression panels and patterns of serum exosomes from three groups, which included healthy, latently infected and actively infected TB individuals. (1) We identified the DEGs and screened the top-20 DEGs from the three groups; (2) we classified all the DEGs into six expression patterns and screened the top-20 genes from each pattern; (3) we identified numerous *Mtb* RNAs in the exosomes of LTBI patients. Our findings not only indicate the selective packaging of RNA cargoes into exosomes under different physiological status but also facilitate the development of potential targets for the diagnosis, prevention, and treatment of tuberculosis.

**FIGURE 6:**
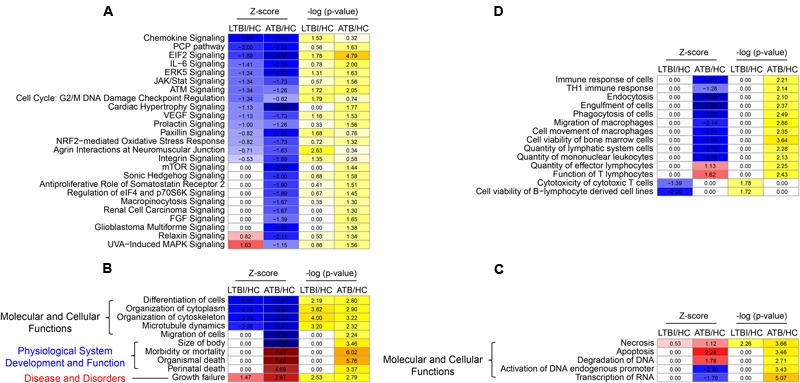
Functional and pathway analyses using Ingenuity Pathway Analysis (IPA) among the three samples. **(A)** The IPA canonical pathway analysis indicating the 25 pathway (*Z*-score ≠ 0 in the LTBI or ATB samples). **(B)** The top-10 significantly changed items (| LTBI *Z*-score| + | ATB *Z*-score|) covered the three IPA categories including the “Molecular and Cellular Functions,” “Physiological System Development and Function,” and “Disease and Disorders.” **(C)** Five selected “Molecular and Cellular Functions” IPA items (*Z*-score ≠ 0 in the LTBI or ATB samples). **(D)** Some immune response related items (*Z*-score ≠ 0 in the LTBI or ATB samples). In addition, *Z*-score represents the IPA regulation trends [*Z*-score > 0: up-regulation (red); *Z*-score < 0: down-regulation (blue)]. Colors indicate the estimate of base 10 log ratio of *P*-values that ranges from white (low) to orange (high) (–Log (*P*-value) > 1.3: high significance level).

To further explore the mechanism of RNA packaging into exosomes under different infectious status, we performed functional and pathway analyses using IPA for the three samples (Supplementary Table 7). The IPA canonical pathway analysis indicated that the 25 signaling pathways were greatly suppressed in ATB samples (the second column of **Figure 6A**). Among these, the *P*-values of 20 items were less than 0.05 (the fourth column of **Figure 6A**). In the LTBI samples, relatively few pathways (15: the first column of **Figure 6A**) were suppressed, and 12 items demonstrated a weaker inhibitory effect than what was observed in the ATB samples. The inhibitory effect on signaling pathways in the exosomes increased with latent TB developing to the active disease, suggesting that different inhibition of cell activities occurs at different stages of *Mtb* infection.

Among all the IPA “Diseases and Bio Functions” items, the top-10 significantly changed items covered the three IPA categories including the “Molecular and Cellular Functions,” “Physiological System Development and Function,” and “Disease and Disorders” (**Figure 6B**). All categories displayed deteriorated trend in the ATB samples (the second column of **Figure 6B**), suggesting a systematic deteriorating state of the ATB patients. However, only five IPA functions (the first column of **Figure 6A**) exhibited a weaker trend in terms of deterioration in the LTBI samples, since most LTBI patients showed no symptoms of disease^2^. Additionally, five other IPA items from the “Molecular and Cellular Functions” category including “necrosis,” “apoptosis,” and “degradation of DNA” exhibited an overall deteriorated profile in the exosomes of ATB samples (the second column of **Figure 6C**), while it was only “necrosis” function that increased slightly in the LTBI samples (the first column of **Figure 6C**).

Cellular and humoral immunity plays a fundamental role in host defense against *Mtb* infection and dissemination (Zuñiga et al., 2012). According to the IPA “Diseases and Bio Functions” analysis, many immune response related items were significantly altered in serum exosome mRNA data (**Figure 6D**). Firstly, four types of items were suppressed in the ATB samples (the second column of **Figure 6D**), including: (1) immune response related functional items: “immune response of cells” and “Th1 immune response” functions, (2) phagocytosis related functional items: “endocytosis,” “engulfment of cells,” and “phagocytosis of cells,” (3) cell movement related functional items: “migration of macrophages” and “cell movement of macrophages,” and (4) other immune cell phenotype related functional items: “quantity of mononuclear leukocytes,” “quantity of lymphatic system cells,” and “cell viability of bone marrow cells functions.” However, all the items in the LTBI samples were consistent with those in the HC samples (the first column of **Figure 6D**). In addition, the “quantity of effector lymphocytes” and “function of T lymphocytes” functions were up-regulated in the ATB samples, and remained unchanged in the LTBI samples. Finally, both the “cytotoxicity of cytotoxic T cells” and “cell viability of B-lymphocyte derived cell lines” functions significantly declined in the LTBI samples but remained constant in the ATB samples (**Figure 6D**).

Overall, our IPA analysis not only provided some potential biomarkers for the diagnosis of latent and active tuberculosis but also reflects some features suggestive of a gradual decline in health status as *Mtb* infection progresses.

## Author Contributions

LL and XZ conceived and designed the experiments. LL, XZ, JW, LP, HJ, and ZL performed the experiments. CL and ND analyzed the data. ZZ, FC, JZ, TC, and XJ provided suggestions on analysis. LL, CL, ND, and XZ wrote the paper, and FC revised the manuscript. All authors read and approved the final manuscript.

## Conflict of Interest Statement

The authors declare that the research was conducted in the absence of any commercial or financial relationships that could be construed as a potential conflict of interest.

## Footnotes

1 https://www.qiagenbioinformatics.com

2 https://www.cdc.gov/tb/publications/ltbi/diagnosis.htm

## Abbreviations

ATB patient: active tuberculosis patient
DEG: differentially expressed gene
IGRA: interferon-gamma release assay
LTBI patient: latent tuberculosis infection patient
NTM: non-tuberculous mycobacteria
TST: tuberculin skin test
