# Supplementary material for: RNA Profiling Analysis of the Serum Exosomes Derived from Patients with Active and Latent Mycobacterium tuberculosis Infection
Source: Front Microbiol. 2017 Jun 12;8:1051. doi: 10.3389/fmicb.2017.01051 (PMC5466984; doi:10.3389/fmicb.2017.01051)
Supplement: Supplementary file 1 [file Table_1.DOCX]

**Supplemental table 1 The sequencing data of the HC,LTBI and ATB samples**

| **Sample** | **Number of reads** | **Number of mapped reads** | **Mapping rate** | **Number of total expressed genes** | **Number of expressed protein coding genes** |
| --- | --- | --- | --- | --- | --- |
| HC 1 | 46838469*2 | 77844786 | 83.10% | 44187 | 18913 |
| HC 2 | 50212267*2 | 83454709 | 83.10% |  |  |
| LTBI 1 | 30449081*2 | 40819459 | 67.00% | 43428 | 18882 |
| LTBI 2 | 39015351*2 | 66816156 | 85.60% |  |  |
| ATB 1 | 41579702*2 | 73203168 | 88.00% | 44261 | 18926 |
| ATB 2 | 38570735*2 | 68297796 | 88.50% |  |  |
