# Supplementary material for: RNA Profiling Analysis of the Serum Exosomes Derived from Patients with Active and Latent Mycobacterium tuberculosis Infection
Source: Front Microbiol. 2017 Jun 12;8:1051. doi: 10.3389/fmicb.2017.01051 (PMC5466984; doi:10.3389/fmicb.2017.01051)
Supplement: Supplementary file 4 [file Table_4.DOCX]

**Supplemental Table 4** Function annotation for 12 DEGs in the ATB samples

| **Gene symbol** | **Location** | **Official full name** | **Gene Ontology function** |
| --- | --- | --- | --- |
| *NTS* | chr12: 85874295- 85882992 | neurotensin | Neuropeptide hormone activity |
| *POSTN* | chr13: 37562583- 37598844 | periostin | Heparin binding  metal ion binding  protein binding |
| *RBM11* | chr21: 14216130- 14228372 | RNA binding motif protein 11 | Poly(U) RNA binding  protein binding  protein homodimerization activity |
| *MMRN1* | chr4: 89879532- 89954629 | multimerin 1 | Calcium ion binding  protein binding |
| *ALDH1L2* | chr12: 105019784- 105084577 | aldehyde dehydrogenase 1 family member L2 | [aldehyde dehydrogenase (NAD) activity](http://amigo.geneontology.org/amigo/term/GO:0004029)  [formyltetrahydrofolate dehydrogenase activity](http://amigo.geneontology.org/amigo/term/GO:0016155)  [hydroxymethyl-, formyl- and related transferase activity](http://amigo.geneontology.org/amigo/term/GO:0016742) |
| *SLC17A2* | chr6: 25912754- 25930726 | solute carrier family 17 member 2 | [sodium:phosphate symporter activity](http://amigo.geneontology.org/amigo/term/GO:0005436) |
| *HMMR* | chr5: 163460203- 163491945 | hyaluronan mediated motility receptor | [hyaluronic acid binding](http://amigo.geneontology.org/amigo/term/GO:0005540)  [protein binding](http://amigo.geneontology.org/amigo/term/GO:0005515) |
| *OFCC1* | chr6: 9596110- 10211608 | orofacial cleft 1 candidate 1 | Protein binding |
| *SLC4A7* | chr3: 27372721- 27484420 | solute carrier family 4 member 7 | [anion transmembrane transporter activity](http://amigo.geneontology.org/amigo/term/GO:0008509)  [inorganic anion exchanger activity](http://amigo.geneontology.org/amigo/term/GO:0005452)  [sodium:bicarbonate symporter activity](http://amigo.geneontology.org/amigo/term/GO:0008510) |
| *CLU* | chr8: 27596917- 27615031 | clusterin | [NOT ATPase activity](http://amigo.geneontology.org/amigo/term/GO:0016887)  [chaperone binding](http://amigo.geneontology.org/amigo/term/GO:0051087)  [misfolded protein binding](http://amigo.geneontology.org/amigo/term/GO:0051787)  [protein binding](http://amigo.geneontology.org/amigo/term/GO:0005515)  [ubiquitin protein ligase binding](http://amigo.geneontology.org/amigo/term/GO:0031625) |
| *VNN2* | chr6: 132743870- 132763459 | Vanin 2 | [pantetheine hydrolase activity](http://amigo.geneontology.org/amigo/term/GO:0017159) |
| *ACSL4* | chrX: 109624244- 109733403 | acyl-CoA synthetase long-chain family member 4 | [ATP binding](http://amigo.geneontology.org/amigo/term/GO:0005524)  [arachidonate-CoA ligase activity](http://amigo.geneontology.org/amigo/term/GO:0047676)  [decanoate--CoA ligase activity](http://amigo.geneontology.org/amigo/term/GO:0102391)  [long-chain fatty acid-CoA ligase activity](http://amigo.geneontology.org/amigo/term/GO:0004467)  [very long-chain fatty acid-CoA ligase activity](http://amigo.geneontology.org/amigo/term/GO:0031957) |
