# Supplementary material for: RNA Profiling Analysis of the Serum Exosomes Derived from Patients with Active and Latent Mycobacterium tuberculosis Infection
Source: Front Microbiol. 2017 Jun 12;8:1051. doi: 10.3389/fmicb.2017.01051 (PMC5466984; doi:10.3389/fmicb.2017.01051)
Supplement: Supplementary file 5 [file Table_5.DOCX]

**Supplemental table 5 The top 20 *Mtb* high expression genes in the LTBI samples.**

| **Function catalogs** | **Gene** | **Reads** | **Products** | **Antigen** |
| --- | --- | --- | --- | --- |
| polyketid and non-ribosomal peptide sythesis | *pks12* | 29 | polyketide synthase | - |
|  | *pks5* | 12 | polyketide synthase | - |
|  | *pks7* | 11 | polyketide synthase | - |
|  | *pks2* | 11 | polyketide synthase | √ |
|  | *nrp* | 11 | unknown non-ribosomal peptide synthase | - |
| PPE family | *PPE56* | 22 | PPE family protein | - |
|  | *PPE8* | 16 | PPE family protein | - |
|  | *PPE55* | 16 | PPE family protein | √ |
|  | *PPE54* | 14 | PPE family protein | √ |
| transpoase | *Rv3023c* | 13 | transposase | - |
|  | *Rv1047* | 13 | transposase | - |
|  | *Rv3115* | 13 | transposase | √ |
|  | *Rv2512c* | 12 | insertion sequence element IS1081 transposase | √ |
|  | *Rv1199c* | 12 | insertion sequence element IS1081 transposase | - |
| lipid biosynthesis | *fas* | 18 | fatty acid synthase | - |
| aspartate family | *asnB* | 13 | asparagine synthase B | - |
| aminoacyl tRNA synthases and their modificaiton | *lysX* | 12 | c-term lysyl-tRNA synthase | - |
| sulphur metabolism | *atsB* | 12 | proable arylsulfatase | - |
| other | *Rv3903c* | 11 | alanine%2Fproline-rich protein | - |
|  | *Rv2566* | 13 | hypothetical protein | - |
