# Supplementary material for: RNA Profiling Analysis of the Serum Exosomes Derived from Patients with Active and Latent Mycobacterium tuberculosis Infection
Source: Front Microbiol. 2017 Jun 12;8:1051. doi: 10.3389/fmicb.2017.01051 (PMC5466984; doi:10.3389/fmicb.2017.01051)
Supplement: Supplementary file 6 [file Table_6.DOCX]

**Supplemental table 6 *Mtb* Genes of LTBI that were known to be detected in protein forms from the other exosome studies*.**

| **Gene** | **Reads** | **Products** | **Antigen** | **Functions** |
| --- | --- | --- | --- | --- |
| *pstS1* | 7 | phosphate ABC transporter substrate-binding lipoprotein PstS | √ | Carbohydrates,oranic acids and alcohols |
| *glnA1* | 6 | glutamine synthetase | √ | Amino acid biosynthesis |
| *garA* | 3 | glycogen accumulation regulator GarA | √ | - |
| *dnaK* | 2.5 | chaperone protein DnaK | √ | chaperones/Heat shock |
| *fba* | 2.5 | fructose-bisphosphate aldolase | - | energy metabolism |
| *icd2* | 2 | isocitrate dehydrogenase | √ | energy metabolism |
| *ctpD* | 2 | cobalt%2Fnickel-exporting P-type ATPase | - | transpotr/binding proteins |
| *sahH* | 2 | adenosylhomocysteinase | - | sulphur metabolism |

* Sinsimer, D., Huet, G., Manca, C., Tsenova, L., Koo, M.-S., Kurepina, N., et al. (2008). The phenolic glycolipid of mycobacterium tuberculosis differentially modulates the early host cytokine response but does not in itself confer hypervirulence. Infect. Immun. **76**, 3027-3036. doi: 10.1128/IAI.01663-07.
