# Supplementary material for: RNA Profiling Analysis of the Serum Exosomes Derived from Patients with Active and Latent Mycobacterium tuberculosis Infection
Source: Front Microbiol. 2017 Jun 12;8:1051. doi: 10.3389/fmicb.2017.01051 (PMC5466984; doi:10.3389/fmicb.2017.01051)
Supplement: Supplementary file 8 [file Image_1.PDF]

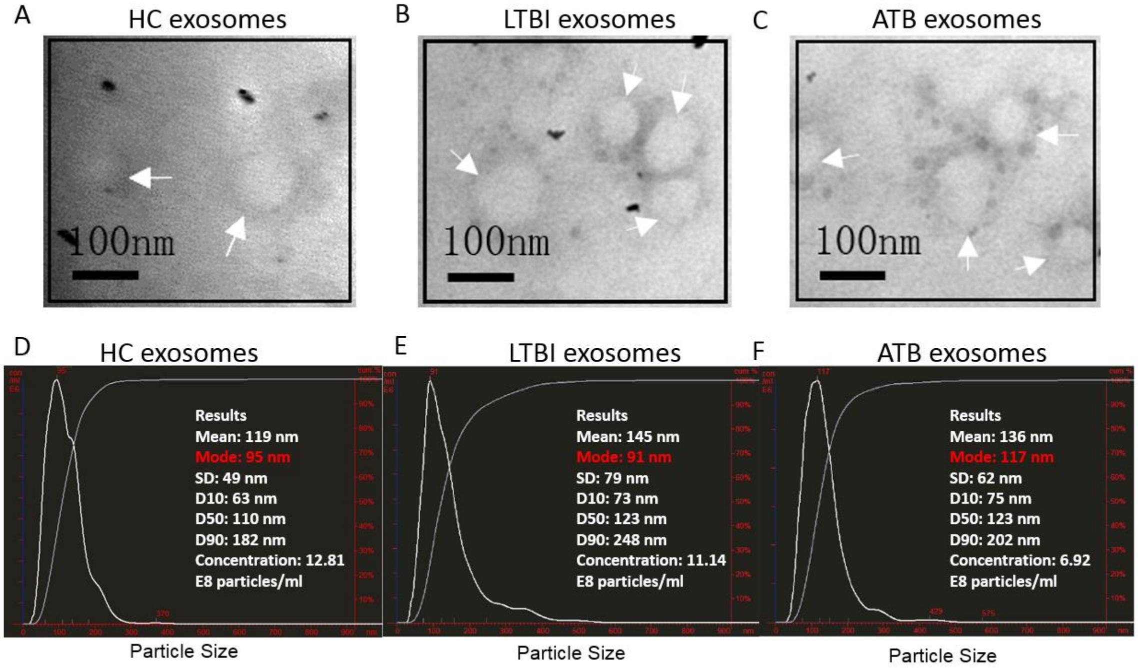

Supplemental figure 1 The TEM images and particle size of the exosomes. (A-C) The transmission electron microscopy images (scale bar =100 nm) of serum exosomes from HC, LTBI and ATB samples. The exosomes were characterized morphologically by transmission electron microscopy. TEM demonstrates successful isolation of exosome-sized particles (~100 nm, white arrows in the figure). (D-F) Nanoparticle tracking analysis (NTA) of the exosome size distribution from HC, LTBI and ATB samples. The NTA results indicate the diameter distributions that peak at 95nm, 91nm, and 117nm for the HC, LTBI and ATB samples.
