# Supplementary material for: RNA Profiling Analysis of the Serum Exosomes Derived from Patients with Active and Latent Mycobacterium tuberculosis Infection
Source: Front Microbiol. 2017 Jun 12;8:1051. doi: 10.3389/fmicb.2017.01051 (PMC5466984; doi:10.3389/fmicb.2017.01051)
Supplement: Supplementary file 9 [file Image_2.PDF]

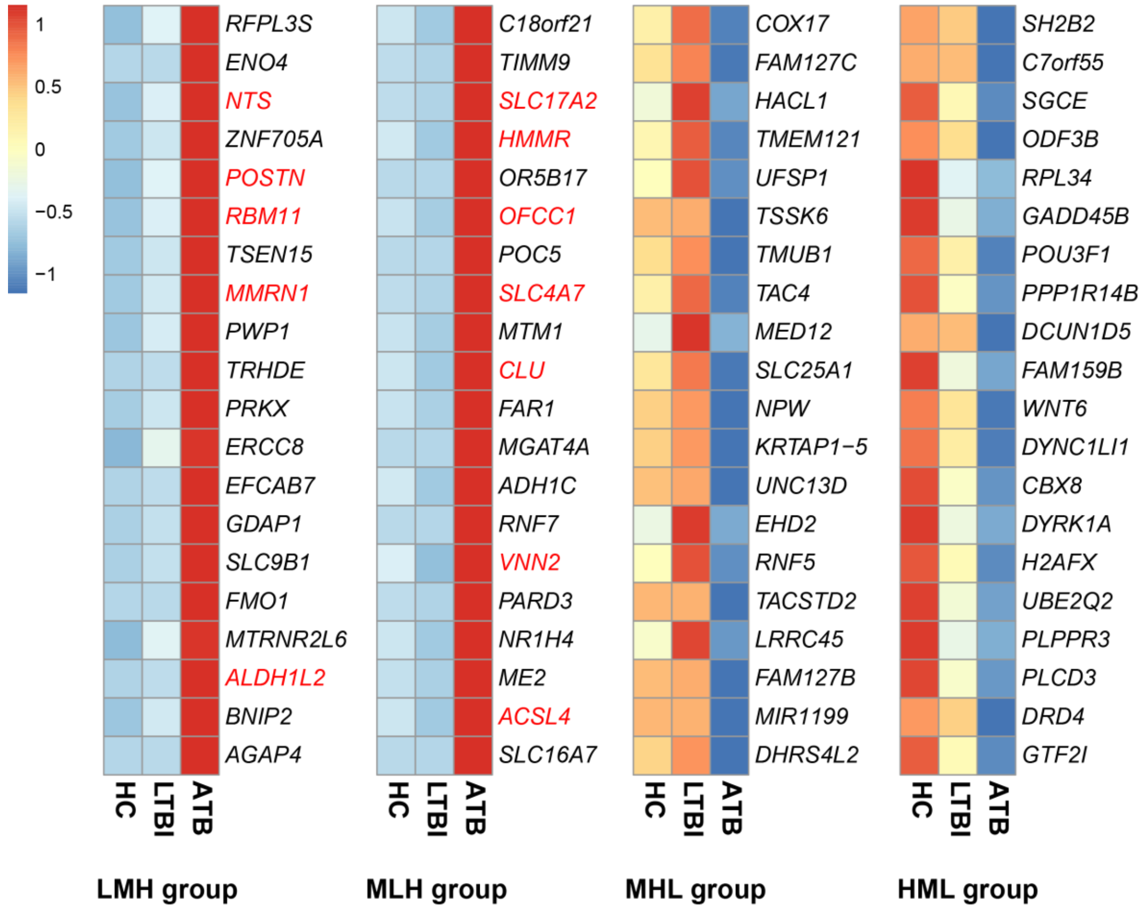

Supplemental figure 2 The top-20 specifically high expressed and low expressed genes from LMH, MLH, MHL and HML patterns in ATB.
